# Prostate cancer incidence among finasteride and alpha-blocker users in the Finnish Prostate Cancer Screening Trial

**DOI:** 10.1038/sj.bjc.6605188

**Published:** 2009-08-04

**Authors:** T J Murtola, T L J Tammela, L Määttänen, M Ala-opas, U H Stenman, A Auvinen

**Affiliations:** 1 School of Public Health, University of Tampere, 33101 Tampere, Finland; 2 Department of Surgery, Central Finland Central Hospital, 40620 Jyväskylä, Finland; 3 Department of Urology, Tampere University Hospital, 33521 Tampere, Finland; 4 Medical School, University of Tampere, 33101 Tampere, Finland; 5 The Finnish Cancer Registry, 00130 Helsinki, Finland; 6 Department of Urology, Helsinki University Hospital, 00029 Helsinki, Finland; 7 Department of Clinical Chemistry, University of Helsinki, 00014 Helsinki, Finland

**Keywords:** alpha-blockers, benign prostatic hyperplasia, finasteride, epidemiology, prostatic neoplasms, screening

## Abstract

**Background:**

The Prostate Cancer Prevention Trial has shown a protective effect of finasteride on prostate cancer in low-risk men. It is uncertain whether similar results can be expected when finasteride is used to treat benign prostatic hyperplasia.

**Methods:**

We performed an observational cohort study within the Finnish Prostate Cancer Screening Trial. Using a comprehensive prescription database on medication reimbursements during 1995–2004 of men using finasteride or alpha-blockers for benign prostatic hyperplasia, we evaluated prostate cancer incidence among 23 320 men screened during 1996–2004.

**Results:**

Compared to medication non-users, overall prostate cancer incidence was not significantly affected in finasteride users (hazard ratio 0.87; 95% CI 0.63–1.19). Incidence of Gleason 2–6 tumours, however, was decreased among finasteride users (HR 0.59; 95% CI 0.38–0.91), whereas incidence of Gleason 7–10 tumours was unchanged (HR 1.33; 95% CI 0.77–2.30). The protective effect concerned mainly screen-detected tumours. Overall prostate cancer risk was not significantly reduced among alpha-blocker users relative to non-users, but decreased incidence of high-grade tumours was observed (0.55; 95% CI 0.31–0.96).

**Conclusions:**

The detection of low-grade, early-stage tumours is decreased among men who use finasteride for symptomatic BPH. The protective effect of finasteride can also be expected in men with benign prostatic hyperplasia.

Finasteride, a 5-alpha reductase enzyme-inhibitor that inhibits conversion of testosterone into active androgen metabolite dihydrotestosterone, thereby lowering prostate volume and serum prostate-specific antigen (PSA) level (Marberger, 2006), is used for treatment of benign prostatic hyperplasia (BPH) and male pattern baldness. The Prostate Cancer Prevention Trial (PCPT) has reported a 25% decrease in prostate cancer incidence in men receiving finasteride compared with placebo (Thompson *et al*, 2003). The trial participants had baseline PSA 3.0 ng ml^−1^ or less and low symptom score of lower urinary tract symptoms (LUTS). The restrictive inclusion criteria limit the generalisability of the findings. It is not known whether the results are applicable to men using finasteride for symptomatic BPH.

*α*1-Adrenoceptor antagonists (alpha-blockers) are used in the medical management of symptoms of benign prostatic hyperplasia. Alpha-blockers lower smooth muscle tension in the prostate and urinary tract, thereby improving urinary flow and decreasing LUTS (Ishizuka *et al*, 2002). Some experimental studies have reported increased prostate cancer cell apoptosis after treatment with quinazoline-derived alpha-blockers, terazosin and doxazosin (Kyprianou and Benning, 2000; Benning and Kyprianou, 2002). One cohort study has reported a decreased incidence among alpha-blocker users (Harris *et al*, 2007).

We evaluated the effect of finasteride and alpha-blocker usage on prostate cancer incidence in a cohort of men participating in the screening arm of the Finnish Prostate Cancer Screening Trial during 1996–2004.

## Materials and methods

The Finnish Prostate Cancer Screening Trial is a part of the European Randomized Study of Prostate Cancer Screening. The trial assesses whether screening can reduce prostate cancer mortality (Määttänen *et al*, 1999) The ethical committees of each participating hospital approved the study protocol. In 1996–1999, all men aged 55–67 years and residing in the metropolitan areas of Helsinki and Tampere (80 484 men) were identified from the population register of Finland and randomly assigned into either the screening arm (32 000 men) or the control arm (48 484 men) of the trial. The detailed protocol has been described previously (Määttänen *et al*, 1999). For exclusion of prevalent prostate cancer cases at randomisation, the cohort was linked to the comprehensive Finnish Cancer Registry (Teppo *et al*, 1994).

Men in the screening arm were recruited with mailed invitations to undergo a PSA screening test at 4-year intervals. After a written informed consent, a blood sample was drawn. All participants also filled in a questionnaire on prostate cancer family history and previous prostatic diseases. Participants of the third screening round were asked to provide information on height and weight for calculation of the body mass index (BMI). The population was linked annually to the Finnish Cancer Registry to obtain information on cases diagnosed between the screening rounds.

During 1996–2004 two screening rounds were completed. The third screening round was started in 2004. A total of 23 320 men (73%) had at least one PSA determination during the study period. A total of 1594 new cases were diagnosed in the screening arm. Of these, 1273 cases were screen detected, whereas 321 were interval cancers. TNM stage was available for 99.6% and Gleason grade in 97.7% of the tumours.

The prescription database of the Social Insurance Institution of Finland (SII) provided detailed information on finasteride and alpha-blocker usage during 1995–2004 for each study participant. SII provides reimbursements for the cost of medicines prescribed by a physician (with the exception of hospital inpatients) to each Finnish citizen as part of the national public health insurance (Martikainen and Rajaniemi, 2002). All reimbursements for purchased prescription drugs approved as reimbursable by the SII are recorded in the reimbursement database.

All drugs in clinical use for treatment of BPH in Finland are reimbursable and available only through a physician's prescription. During the study period, these included 5*α*-reductase inhibitor, finasteride, and alpha-blockers, tamsulosin (since 1996) and alfuzosin (since 1997). Finasteride prescribed for treatment of androgenic alopecia was not reimbursable and thus not recorded by the prescription database.

Amount of medication use was defined as daily doses in treatment of BPH: finasteride 5 mg, tamsulosin 0.4 mg and alfuzosin 10 mg per day. The cumulative number of daily doses during the study period was calculated for each person based on dosage, package size and number of packages bought each year. Cumulative amounts of tamsulosin and alfuzosin use were summed to obtain total usage of alpha-blockers.

### Statistical analysis

Cox proportional hazards regression was used to estimate hazard ratios and their 95% confidence intervals (CIs) for prostate cancer by medication usage. Men who used neither finasteride nor alpha-blockers were used as the reference group in all analyses on risk.

Each man in the study population contributed person time from the date of the first screening test until date of diagnosis, emigration from the study area, death or end of study period (31 December 2004), whichever came first.

Time-dependent Cox regression model with adjustment for age (continuous time-dependent covariate; *P*<0.001 in the model), family history (father, brother or son diagnosed with prostate cancer; *P*=0.916), simultaneous use of the other group of BPH drugs (*P*=0.07 for finasteride or *P*=0.847 for alpha-blockers), number of PSA screens attended (continuous time-dependent covariate; *P*<0.001) and the calendar period of screening test (before or after year 2000; *P*=0.209) was used for analyses. Additional adjustment for BMI (*P*=0.854) and prostate volume (as measured by a urologist in a transrectal ultrasound examination; *P*=0.047) was used in subgroup analyses of men with this information available (3130 men attending the third screening round in 2004). Age was the single most influential covariate in the model.

Each man, who was not a medication user at randomisation, contributed person time in the analysis as a non-user until the first medication reimbursement. After a period of 6 months without reimbursements, the men were reclassified as medication non-users. Exposure status was allowed to change as often as necessary. If the man had simultaneously used both finasteride and alpha-blockers, he contributed person time (and potentially events) in both categories, that is, as a finasteride user and as an alpha-blocker user.

Amount (daily doses) and duration (in years) of medication use were analysed as time-dependent covariates. In analyses stratified by cumulative amount/duration of medication use, the users contributed person time in lower stratum until reaching the cut-point for upper stratum.

Trends in incidence by amount or duration of medication use were tested by adding these indicators into Cox regression model as continuous covariates.

The proportional hazards assumption was tested by adding the interaction term for finasteride or alpha-blocker use and person time to the model. The term was not statistically significant by the likelihood ratio test, confirming the assumption.

All analyses were performed using SPSS 15.0 statistical software.

## Results

Of the 23 320 men in the cohort, 1754 (7.5%) had used finasteride and 3848 (16.5%) had used either tamsulosin or alfuzosin. Prevalence of medication use increased with age at start of follow-up. Family history was comparable in the two groups (Table 1). The age-standardised median PSA was higher among BPH medication users compared with non-users (Table 1). Both finasteride and alpha-blocker use was associated with a decreased proportion of free PSA, the effect again being stronger in finasteride users. Among the men attending the third screening round, average prostate volumes and the median BMI were higher among medication users than non-users (Table 1).

Overall, finasteride use was not significantly associated with risk (HR 0.87, 95% CI 0.63–1.19; Table 2). However, the risk of low-grade (Gleason 2–6) tumours was decreased among finasteride users (HR 0.59; 95% CI 0.38–0.91) and further diminished in relation to the cumulative amount and duration of medication usage (*P* for trend 0.009 and 0.019, respectively; Table 2). Generally, incidence of high-grade, organ-confined or advanced stage tumours was not affected by finasteride usage (Table 2). However, among long-term finasteride users, increased incidence of high-grade tumours was observed (HR 2.49; 95% CI 1.27–4.89 for men who had used at least 1087 doses of finasteride). Overall risk did not differ between alpha-blocker users and non-users. However, lowered incidence of high-grade tumours was observed (HR 0.55; 95% CI 0.31–0.96), with a decreasing trend in risk with cumulative duration of alpha-blocker use (Table 3).

In an analysis stratified by serum PSA concentration, prostate cancer risk was decreased in finasteride and alpha-blocker users with PSA⩾4 ng ml^−1^ (the cut-off value for screen-positive test, i.e., indication for prostate biopsy; Table 4). The point estimate was lower among finasteride users, but the confidence intervals overlap. The decreased risk was driven by the lower incidence of screen-detected tumours among these men. Risk of interval cancers, that is, tumours diagnosed between the screening rounds, was not significantly affected in finasteride users. However, among alpha-blocker users with PSA below 4 ng ml^−1^, the risk of interval cancer was increased (HR 2.46; 95% CI 1.21–5.00).

## Discussion

In our cohort study within the screening arm of the Finnish Prostate Cancer Screening Trial we found a reduced risk of low-grade prostate cancer among finasteride users, among whom an increased risk of high-grade cancers was seen among long-term users. These findings confirm previous findings on this topic, but provide wider generalisability than the Prostate Cancer Prevention Trial, and improved internal validity compared with non-randomised studies due to comprehensive and systematic case ascertainment. Alpha-blocker usage generally did not affect incidence, but some evidence for a decreased risk of high-grade tumours was observed.

Availability of comprehensive and detailed information on medication purchases from the SII prescription database allowed us to evaluate BPH medication usage accurately and in an unbiased fashion. Finasteride, tamsulosin and alfuzosin were available in Finland only through the physician's prescription during the study period, so their purchase is comprehensively documented by the prescription database.

Our finding of a decreased risk of low-grade tumours among finasteride users is similar with the results from the Prostate Cancer Prevention Trial (Thompson *et al*, 2003). However, a major limitation of our study in comparison to PCPT is that this is a non-experimental study without any intervention related to BPH medication, whereas PCPT was a randomised clinical trial. Due to lack of random allocation, our results are, therefore, more prone to systematic differences between men with or without BPH. In our study, finasteride was prescribed for treatment of symptomatic BPH, whereas men eligible for PCPT were free of LUTS and had PSA of 3 ng ml^−1^ or less (Thompson *et al*, 2003). Therefore, our study population was more representative of the general population with BPH in terms of prostate volume and PSA.

The PCPT study protocol included offering an end-of-study prostate biopsy for all willing participants regardless of symptoms or PSA level, resulting in high prostate tumour incidence with obvious potential for overdiagnosis of indolent tumours but also reducing possibility of detection bias (Thompson *et al*, 2003). In our study, all men were screened and only screen-positive men (PSA 4 ng ml^−1^ or higher or PSA between 3 and 4 ng ml^−1^ and proportion of free PSA below 16%) underwent biopsy. Thus, our study shows that the protective effect also applies to men using finasteride for treatment of BPH and attending standard urological care, a conclusion supported by a previous case–control study (Irani *et al*, 2002). Unlike PCPT, we did not observe a significant decrease in overall risk among finasteride users, although the relative risk reduction in our study (22%) was close to that reported in the PCPT (25%). However, among the biopsied (screen-positive) men, the overall risk decrease was also significant in our study. It should be noted that the average duration and cumulative amount of finasteride usage was lower in our study than in the PCPT.

In this study, finasteride users had symptomatic BPH, and confounding by indication could affect the results, if BPH affects the risk of prostate cancer or additional testing in the clinical setting would affect prostate cancer detection. In this case, a positive association between BPH and prostate cancer and further PSA tests would be expected to increase detection. In our study, the contrary was observed, so BPH as indication for finasteride use cannot account for our findings. Unlike the PCPT trial (Thompson *et al*, 2003), we did not observe overall risk increase for high-grade prostate tumours among finasteride users. However, the risk was increased among long-term users, although no dose dependence between cumulative dose or duration of finasteride use and risk of high-grade cancer was observed. Later analyses of the PCPT results have shown that the observed higher proportion of high-grade cancers in finasteride-treated men is due to detection bias caused by decreased prostate volume, increased sensitivity of PSA to detect prostate cancer and altered tumour grading in finasteride users (Thompson *et al*, 2006; Lucia *et al*, 2007; Pinsky *et al*, 2008). This effect could also have caused the slightly increased incidence of high-grade tumours in our study.

Use of alpha-blockers tamsulosin and alfuzosin had no effect on overall risk, but there was some indication of a reduced risk of high-grade tumours. Previously, quinazoline-derived alpha-blockers, terazosin and doxazosin, have been reported to inhibit prostate cancer cell growth and reduce incidence (Kyprianou and Benning, 2000; Benning and Kyprianou, 2002; Harris *et al*, 2007). Our results suggest that tamsulosin and alfuzosin could have similar effects but this aspect needs further research.

PCPT reported decreased serum PSA concentrations in finasteride users as compared with non-users (Etzioni *et al*, 2005). In our study, instead, serum PSA was increased in both finasteride and alpha-blocker users. Odds of having serum PSA exceeding the prostate biopsy cut-point (4 ng ml^−1^) was not decreased, but conversely increased in finasteride users (OR 2.37; 95% CI 2.13–2.64). This is due to the fact that in our study these medications were used for BPH treatment and men using them are not comparable with non-users. Therefore, these differences reflect the effect of BPH, and not medications. However, the PSA concentration tended to decrease with increasing cumulative amount of finasteride use, although the geometric mean PSA remained above non-users even among men in the highest quartile of finasteride use (1087 doses or more), though the confidence intervals were wide (Table 1). A similar association was observed with increasing duration of finasteride use (results not shown). For alpha-blockers, the geometric mean PSA was constantly higher among users than non-users with no decrease by duration or amount of use. The decrease in the proportion of free PSA was more pronounced in finasteride users and increased in relation with amount and duration of medication use, probably reflecting its effect on proportion of free PSA in long-term use, as such relation was not observed in alpha-blocker users.

Both finasteride and alpha-blocker use was associated with a decreased risk of screen-detected tumours among screen-positive men. As the risk decrease was observed among users of both drug groups, it may be due to the underlying disease, BPH. PSA elevation in men with LUTS (medication users) is often caused by prostate enlargement, whereas in men with no such symptoms (medication non-users) PSA increase is more commonly caused by a prostate cancer. Alpha-blocker users, whose PSA was below 4 ng ml^−1^, were at increased risk between the screening rounds. This finding is consistent with our results from the previous case–control study (Murtola *et al*, 2007) and may reflect a similar mechanism. The Finnish guideline for clinical management of BPH recommends using alpha-blockers if a man has significant LUTS but no prostate enlargement (Finnish Medical Society Duodecim). Symptoms lead to clinical examinations and thus possibly to diagnosis despite the negative screening test. Additionally, men with significant LUTS may undergo transurethral resection of the prostate, in which incidental prostate cancer is a common finding (Merrill and Wiggins, 2002). This would lead to a bias of greater cancer detection in alpha-blocker users but not in finasteride users, as finasteride reduces the need for surgical management of BPH (Roehrborn *et al*, 2004).

We were able to control the confounding caused by age and familial predisposition (Crawford, 2003) in the analysis. Confounding by ethnicity (Crawford, 2003) is likely minimal due to the homogeneity of the Finnish population with over 98 percent of the population being of Finnish ancestry (Statistics Finland). Additionally, we had information on prostate volume and BMI for a proportion of our study population. Adjustment for these variables did not materially affect the results.

Our study has some limitations. The number of stage T_3_, stage T_4_, lymph node-positive or metastatic tumours was small in our study population of screened men, limiting our inference concerning the risk of advanced cancer. Similarly, we could not analyse mortality among finasteride users due to the small number of deaths.

We did not have information on less established prostate cancer risk factors such as dietary patterns or nutrient intake (such as selenium or vitamin E). Medication users may be more health conscious than non-users, and follow a healthier diet, which could have reduced the incidence in medication users.

Some exposure misclassification was likely caused by the fact that the cohort follow-up started in 1996 at the earliest, though finasteride was licensed in Finland in 1992. Additionally, SII does not reimburse finasteride prescribed for treatment of androgenic alopecia, and thus we did not have information on finasteride use for this indication. Therefore, some of the finasteride users likely have longer history of use than appeared in our study, a bias that may have weakened the observed association with prostate cancer risk.

The decreased risk among finasteride users in a cohort of men participating in the Finnish Prostate Cancer Screening Trial suggest that finasteride has a clinically significant preventive effect against low-grade tumours also when used for treatment of symptomatic benign prostatic hyperplasia. Future research should aim to evaluate whether finasteride can reduce mortality.

## Figures and Tables

**Table 1:** Characteristics of users and non-users of finasteride and alpha-blockers in the Finnish Prostate Cancer Screening Trial

	**Finasteride usage**		**Alpha-blocker usage^a^**	
	**Yes**	**No**	**Yes**	**No**
Characteristics:				
Participants (*n*)	1754	21 566	3848	19 472
				
*Age at randomisation (years)*	No. of men (% of total)		No. of men (% of total)	
55	261 (3.5)	7295 (96.5)	790 (10.5)	6766 (89.5)
59	427 (6.9)	5804 (93.1)	998 (16.0)	5233 (84.0)
63	476 (9.2)	4674 (90.8)	1025 (19.9)	4125 (80.1)
67	590 (13.5)	3793 (86.5)	1035 (23.6)	3348 (76.4)
Prevalence of family history of prostate cancer (%)^b^	0.3	0.3	0.4	0.3
Mean no. of screening rounds attended	2.0	1.9	2.0	1.9
Geometric mean of PSA (95% CI)^c^	1.58 (0.24–11.05)	1.22 (0.27–7.72)	1.60 (0.28–9.46)	1.22 (0.26–7.67)
	*P*<0.001^d^		*P*<0.001	
				
*Cumulative amount of medication use* e	Non-users	1.22 (0.27–7.72)	Non-users	1.22 (0.26–7.67)
1st quartile	1.95 (0.31–12.94)	—	1.54 (0.26–8.64)	—
2nd quartile	1.72 (0.19–9.56)	—	1.66 (0.19–9.07)	—
3rd quartile	1.49 (0.23–8.94)	—	1.58 (0.29–9.58)	—
4th quartile	1.31 (0.17–1.25)	—	1.80 (0.36–11.66)	—
Geometric mean of % free PSA (95% CI)^c^	22.37 (9.49–48.36)	26.48 (10.40–52.80)	25.26 (10.56–50.96)	26.43 (10.30–52.80)
	*P*<0.001		*P*<0.001	
				
*Cumulative amount of medication use* e	Non-users	1.22 (0.27–7.72)	Non-users	1.22 (0.26–7.67)
1st quartile	22.87 (10.96–45.40)	—	25.26 (10.50–50.99)	—
2nd quartile	22.67 (9.13–47.26)	—	24.66 (9.90–47.47)	—
3rd quartile	21.22 (9.35–48.59)	—	25.36 (10.85–54.85)	—
4th quartile	21.12 (7.75–43.57)	—	25.76 (10.76–52.53)	—
				
*Men attending the third screening round (*N=*3130)*				
Median body mass index	26.7	26.2	26.6	26.2
	*P*=0.032		*P*=0.035	
Median prostate volume (ml)^f^	49	36	42	36
	*P*<0.001		*P*<0.001	

a Includes users of tamsulosin and alfuzosin.

b Father, brother or son diagnosed with prostate cancer prior to initiation of the Finnish Prostate Cancer Screening Trial.

c Age-standardised values.

d *P* estimated using Man–Whitney *U*-test.

e Quartiles for finasteride users : 28–180 doses (1st quartile), 181–398 doses (2nd quartile), 399–1086 doses (3rd quartile), 1087 doses or more (4th quartile); for alpha-blockers: 10–60 doses (1st quartile), 61–180 doses (2nd quartile), 181–629 doses (3rd quartile) and 630 doses or more (4th quartile).

f As measured by a urologist on a transrectal ultrasound examination.

**Table 2:** Hazard ratio for prostate cancer by amount and duration of use of finasteride and by prostate cancer stage and grade, Finnish Prostate Cancer Screening Trial

	**Overall**		**Gleason⩽6**		**Gleason 7–10**		**Organ-confined tumours^a^**		**Advanced tumours^b^**	
**Quantity/duration of medication use**	**No. of cases**	**HR (95% CI)^c^**	**No. of cases**	**HR (95% CI)**	**No. of cases**	**HR (95% CI)**	**No. of cases**	**HR (95% CI)**	**No. of cases**	**HR (95% CI)**
*Finasteride*										
Non-users	1507	Reference	1139	Reference	338	Reference	1364	Reference	143	Reference
All users	87	0.87 (0.63–1.19)	55	0.59 (0.38–0.91)	26	1.33 (0.77–2.30)	81	0.89 (0.65–1.24)	6	0.55 (0.14–2.24)
										
*Cumulative quantity of finasteride use (daily doses)* d										
28–180	34	1.34 (0.74–2.42)	24	0.80 (0.33–1.92)	6	1.17 (0.29–4.74)	32	1.32 (0.70–2.46)	2	1.48 (0.21–10.68)
181–398	21	0.91 (0.50–1.65)	14	0.76 (0.36–1.60)	5	0.79 (0.20–3.20)	19	1.00 (0.55–1.81)	2	—
399–1086	17	0.57 (0.27–1.19)	13	0.64 (0.29–1.43)	4	0.37 (0.05–2.68)	17	0.61 (0.29–1.28)	0	—
⩾1087	15	0.82 (0.47–1.46)	4	0.28 (0.09–0.87)	11	2.49 (1.27–4.89)	13	0.81 (0.45–1.48)	2	0.96 (0.13–6.94)
*P*_trend_ e		0.204		0.009		0.114		0.275		0.415
										
*Years of finasteride use* d										
1	41	0.89 (0.5–1.48)	30	0.62 (0.31–1.24)	7	0.57 (0.14–2.32)	39	0.91 (0.53–1.54)	2	0.66 (0.09–4.71)
2	19	0.96 (0.50–1.85)	13	0.84 (0.38–1.88)	5	1.02 (0.25–4.13)	19	1.03 (0.53–1.99)	0	—
3–4	11	0.72 (0.39–1.35)	7	0.48 (0.20–1.16)	4	1.60 (0.66–3.91)	10	0.70 (0.36–1.34)	2	1.10 (0.15–7.94)
>4	16	1.00 (0.47–2.11)	5	0.40 (0.10–1.61)	10	2.61 (1.06–6.45)	13	1.07 (0.51–2.28)	2	—
*P*_trend_		0.411		0.019		0.057		0.524		0.429

a Men with T_1_N_0/X_M_0/X_ and T_2_N_0/X_M_0/X_ tumours combined.

b Men with stage T_3_N_0/X_M_0/X_, T_4_N_0/X_M_0/X_, T_1–4_N1M0 or T_1–4_N_0–1_M1 tumours combined.

c From Cox proportional hazard regression adjusted for age, family history of prostate cancer, use of alpha-blockers, number of PSA screens and time period of screening (before or after year 2000).

d Stratification in quartiles of cumulative quantity/duration of finasteride use.

e Estimated by including cumulative dose (DDDs) or duration (years) of medication use into Cox regression model as a continuous covariate. All statistical trends are inverse, i.e., indicating a decreased risk with larger amount of medication use.

**Table 3:** Hazard ratio for prostate cancer by amount and duration of use of alpha-blockers and by prostate cancer stage and grade, Finnish Prostate Cancer Screening Trial

	**Overall**		**Gleason⩽6**		**Gleason 7–10**		**Organ-confined tumours^a^**		**Advanced tumours^b^**	
**Quantity/duration of medication use**	**No. of cases**	**HR (95% CI)^c^**	**No. of cases**	**HR (95% CI)**	**No. of cases**	**HR (95% CI)**	**No. of cases**	**HR (95% CI)**	**No. of cases**	**HR (95% CI)**
*Alpha-blockers*										
Non-users	1399	Reference	1041	Reference	330	Reference	1262	Reference	137	Reference
All users	195	1.05 (0.85–1.31)	153	1.20 (0.94–1.52)	34	0.55 (0.31–0.96)	183	1.09 (0.87–1.36)	12	0.70 (0.28–1.73)
										
*Cumulative quantity of alpha-blockers use (daily doses)* d										
10–60	77	1.25 (0.83–1.87)	62	1.65 (1.09–2.49)	12	0.21 (0.03–1.52)	70	1.27 (0.83–1.93)	7	1.17 (0.29–4.72)
61–180	46	1.00 (0.64–1.56)	35	0.84 (0.48–1.49)	8	0.95 (0.39–2.30)	44	0.99 (0.62–1.58)	2	1.14 (0.28–4.64)
181–629	39	1.11 (0.75–1.64)	30	1.21 (0.77–1.88)	8	0.64 (0.24–1.72)	37	1.16 (0.78–1.73)	2	0.54 (0.08–3.86)
⩾630	33	0.89 (0.59–1.36)	26	1.12 (0.72–1.75)	6	0.40 (0.13–1.25)	32	0.96 (0.64–1.46)	1	—
*P*_trend_ e		0.975		0.345		0.053		0.700		0.230
										
*Years of alpha-blockers use* d										
1	111	1.00 (0.73–1.38)	86	1.08 (0.75–1.55)	19	0.60 (0.27–1.35)	102	1.00 (0.72–1.39)	9	1.10 (0.41–2.99)
2	43	1.46 (1.00–2.15)	36	1.67 (1.09–2.56)	5	0.60 (0.19–1.89)	42	1.53 (1.03–2.26)	2	0.70 (0.10–5.01)
3–4	23	0.87 (0.55–1.37)	15	1.04 (0.63–1.70)	8	0.48 (0.15–1.52)	21	0.93 (0.59–1.47)	1	—
>4	18	0.88 (0.42–1.86)	16	1.15 (0.51–2.60)	2	0.38 (0.05–2.73)	18	0.96 (0.45–2.03)	0	—
*P*_trend_		0.858		0.186		0.044		0.580		0.208

a Men with T_1_N_0/X_M_0/X_ and T_2_N_0/X_M_0/X_ tumours combined.

b Men with stage T_3_N_0/X_M_0/X_, T_4_N_0/X_M_0/X_, T_1–4_N1M0 or T_1–4_N_0–1_M1 tumours combined.

c From Cox proportional hazard regression adjusted for age, family history of prostate cancer, use of alpha-blockers, number of PSA screens and time period of screening (before or after year 2000).

d Stratification in quartiles of cumulative quantity/duration of alpha-blocker use.

e Estimated by including cumulative dose (DDDs) or duration (years) of medication use into Cox regression model as a continuous covariate. All statistical trends are inverse, i.e., indicating a decreased risk with larger amount of medication use.

**Table 4:** Hazard ratio for screen-detected and interval prostate cancer among finasteride and alpha-blocker users, stratified by serum PSA level and serum PSA, Finnish Prostate Cancer Screening Trial

	**Overall**		**Screen-detected cancer**		**Interval cancer**	
**Quantity/duration of medication use**	**No. of cases (users/non-users)**	**HR (95% CI)^a^**	**No. of cases**	**HR (95% CI)**	**No. of cases**	**HR (95% CI)**
*Finasteride*						
Serum PSA<4	19/214	0.87 (0.43–1.76)	5/134	0.64 (0.23–1.79)	14/80	1.26 (0.48–3.31)
Serum PSA⩾4	68/1293	0.62 (0.46–0.83)	46/1084	0.61 (0.44–0.84)	22/209	0.49 (0.23–1.06)
						
*Alpha-blockers*						
Serum PSA<4	48/185	1.75 (1.08–2.82)	20/119	1.40 (0.73–2.68)	28/66	2.46 (1.21–5.00)
Serum PSA⩾4	147/1214	0.72 (0.58–0.90)	97/1033	0.66 (0.51–0.84)	50/181	1.06 (0.64–1.73)

a From Cox proportional hazard regression adjusted for age, family history of prostate cancer, finasteride or alpha-blocker use, number of PSA screens and time period of screening (before or after year 2000).
